# P53 germline mutations in childhood cancers and cancer risk for carrier individuals

**DOI:** 10.1054/bjoc.2000.1167

**Published:** 2000-05-18

**Authors:** A Chompret, L Brugières, M Ronsin, M Gardes, F Dessarps-Freichey, A Abel, D Hua, L Ligot, M-G Dondon, B Bressac-de Paillerets, T Frébourg, J Lemerle, C Bonaïti-Pellié, J Feunteun

**Affiliations:** Départment d’Oncologie Pédiatrique, Laboratoire de Génétique Oncologique, CNRS UMR #1599; Unité de Recherches en Epidémiologie des Cancers INSERM U521; Laboratoire de Diagnostic Moléculaire, Départment de Biologie Clinique, Institute Gustave-Roussy, 39 rue Camille Desmoulins, Villejuif Cedex, 94805, France; Laboratoire de Génétique Moléculaire, CHU, Rouen, France

**Keywords:** germline, p53 mutation, cancer risk

## Abstract

The family history of cancer in children treated for a solid malignant tumour in the Paediatric Oncology Department at Institute Gustave-Roussy, has been investigated. In order to determine the role of germline p53 mutations in genetic predisposition to childhood cancer, germline p53 mutations were sought in individuals with at least one relative (first- or second-degree relative or first cousin) affected by any cancer before 46 years of age, or affected by multiple cancers. Screening for germline p53 mutation was possible in 268 index cases among individuals fulfilling selection criteria. Seventeen (6.3%) mutations were identified, of which 13 were inherited and four were de novo. Using maximum likelihood methods that incorporate retrospective family data and correct for ascertainment bias, the lifetime risk of cancer for mutation carriers was estimated to be 73% for males and nearly 100% for females with a high risk of breast cancer accounting for the difference. The risk of cancer associated with such mutations is very high and no evidence of low penetrance mutation was found. These mutations are frequently inherited but de novo mutations are not rare. © 2000 Cancer Research Campaign


*British Journal of Cancer* (2000) **82**(12), 1932–1937
© 2000 Cancer Research Campaign
doi: 10.1054/ bjoc.2000.1167, available online at http://www.idealibrary.com on IDEAL®

### P53 germline mutations in childhood cancers and cancer risk for carrier individuals

**A Chompret[1,3], L Brugières[1], M Ronsin[2], M Gardes[2], F Dessarps-Freichey[2], A Abel[1,3], D Hua[3], L Ligot[3], M-G Dondon[3], B Bressac-de Paillerets[4], T Frébourg[5], J Lemerle[1], C Bonaïti-Pellié[3] and J Feunteun[2]**

[1]Départment d'Oncologie Pédiatrique, [2]Laboratoire de Génétique Oncologique, CNRS UMR #1599, [3]Unité de Recherches en Epidémiologie des Cancers INSERM U521, [4]Laboratoire de Diagnostic Moléculaire, Départment de Biologie Clinique, Institute Gustave-Roussy, 39 rue Camille Desmoulins, 94805 Villejuif Cedex, France; [5]Laboratoire de Génétique Moléculaire, CHU, Rouen, France

**Summary** The family history of cancer in children treated for a solid malignant tumour in the Paediatric Oncology Department at Institute Gustave-Roussy, has been investigated. In order to determine the role of germline p53 mutations in genetic predisposition to childhood cancer, germline p53 mutations were sought in individuals with at least one relative (first- or second-degree relative or first cousin) affected by any cancer before 46 years of age, or affected by multiple cancers. Screening for germline p53 mutation was possible in 268 index cases among individuals fulfilling selection criteria. Seventeen (6.3%) mutations were identified, of which 13 were inherited and four were de novo. Using maximum likelihood methods that incorporate retrospective family data and correct for ascertainment bias, the lifetime risk of cancer for mutation carriers was estimated to be 73% for males and nearly 100% for females with a high risk of breast cancer accounting for the difference. The risk of cancer associated with such mutations is very high and no evidence of low penetrance mutation was found. These mutations are frequently inherited but de novo mutations are not rare. © 2000 Cancer Research Campaign

**Keywords:** germline; p53 mutation; cancer risk

Genetic predisposition to childhood cancer has been borne out by retinoblastoma which proved to be a paradigm for hereditary cancers (Knudson, 1971). Li–Fraumeni syndrome (LFS), a multiple cancer syndrome characterized by a wide spectrum of neoplasms, documented a further hereditary cancer condition likely to affect children (Li et al, 1988; Varley et al, 1997*b*). The definition of LFS proposed by Li and Fraumeni specifies that the proband is diagnosed as having sarcoma before 45 years of age, and also has a first-degree relative with cancer before 45 years of age and another first- or second-degree relative with any cancer diagnosed during this age interval or sarcoma occurring at any age. The most prevalent malignancies observed in LFS include: soft tissue sarcoma, osteosarcoma, brain tumours, adrenocortical carcinoma, leukaemia and breast cancer. Germline mutations in the p53 tumour suppressor gene have been associated with a predisposition to these cancers in families affected by LFS (Malkin, 1994). Fifty to 70% of LFS families display a p53 mutation (Brugières et al, 1993; Birch et al, 1994; Frébourg et al, 1995; Varley et al, 1997*a*), which signifies that mutation screening may have overlooked alterations that affect regulatory regions and not p53 coding sequences and/or that germline mutations of other gene(s) may be responsible for LFS. Germline p53 mutations have also been detected in families presenting tumours outside the spectrum typifying LFS (Malkin et al, 1992; Toguchida et al, 1992). Studies on individuals with typical LFS tumours but not previously selected on family history, have yielded frequencies of germline p53 mutation that vary with age and tumour types (Varley et al, 1999). None of these studies permitted an estimation of cancer risk in mutation carriers, although some unaffected carrier relatives may be found in family studies. Indeed, selection criteria for LFS are so stringent that correction for selection bias is impossible. Even looser criteria, such as Li–Fraumeni-like (LFL) (Eeles, 1995) or Li–Fraumeni incomplete (LFI) (Brugières et al, 1993) do not allow correction for ascertainment bias. This is why we undertook a study at the Institute Gustave Roussy with loose criteria which offer two advantages: (i) they do not imply the existence of highly penetrant susceptibility genes and therefore do not necessarily prohibit the detection of mutations associated with low cancer risk, (ii) correction for selection bias is possible for the estimation of cancer risks in individuals.

*Received 13 May 1999*
*Revised 27 December 1999*
*Accepted 16 February 2000*

*Correspondence to:* J Feunteun

### MATERIALS AND METHODS

### Patients, family history and blood samples

The family history of cancer of children under 18 years treated for all types of solid malignant tumours in the Department of Paediatric Oncology at the Institute Gustave Roussy in Villejuif (France) has been investigated since January 1991. To minimize possible bias due to genetic and environmental heterogeneity, non-white children were excluded. Information was collected through a direct interview with a trained counsellor from families of patients treated in the Department after 1991. Information was obtained via a mailed questionnaire and completed in most cases by a telephone interview, for patients treated before that period but who were no longer followed up.

Family data were collected through the proband's parents. They included information on each of the proband's first- and second-degree relatives and first cousins. When necessary, additional family members, previously informed by the proband's parents, were contacted for a telephone interview. Information on relatives included general characteristics (sex, date of birth, malformations, date and cause of death) and the occurrence of any cancer. When cancers had occurred, confirmation of the diagnoses and age at onset were sought in medical and pathology records. Only invasive cancers were considered, excluding non-melanoma skin cancer and in situ carcinoma. Informed consent was obtained from parents before inclusion in the study.

A subgroup of children with a putative increased frequency of susceptibility genes, was defined on the basis of the occurrence of either of the following criteria: (i) at least one cancer case affecting a first- or second-degree relative or a first cousin before the age of 46 ('familial cases'), (ii) multiple primary cancers in the proband regardless of their family history ('multiple tumour cases').

Blood samples were obtained from the proband, his/her parents and, when a relative was affected, from all the available family members in that branch of the pedigree. Specific informed consent for blood sampling was obtained from all participating relatives. This study received the approval at the local ethics committee (CCPPRB, Kremlin-Bicêtre).

### Genotyping p53

p53 was genotyped in peripheral lymphocytes isolated from fresh blood samples. Direct sequencing was used for the first set of 100 samples. Genomic DNA was amplified as three fragments including respectively exons 2–4, exons 5–8 and exons 9–11. Polymerase chain reaction (PCR) primers sequences are available on request. Genotyping was subsequently carried out with a functional assay in yeast (FASAY), as described by Ishioka et al, (1993), and modified by Flaman et al (1995). Vent DNA polymerase (New England Biolabs) was used to amplify p53 reverse transcripts before transfection in yeast. Yeast colonies carrying a p53 mutant allele were identified either as His-auxotroph, or as red colonies. p53 cDNA was extracted from mutant colonies and sequenced. The FASAY has been reported to reveal over 90% of p53 mutant alleles (Flaman et al, 1995) as did direct sequencing of amplified p53 exons scores in our hands.

### Estimation of cancer risk

The risk of cancer for individuals carrying a p53 mutation was estimated using ARCAD, a maximum likelihood method and a program based on a survival analysis approach in which the event considered is age at onset of cancer (Le Bihan et al, 1995). For each individual, the information used is the genotype (carrier or not), and the phenotype which includes the affection status and the age of onset for affected individuals and the age at examination for unaffected ones. The likelihood L for a sample of n families is the product of likelihoods of each family f:

$$L = \prod_{f=1}^{n} L_f$$

To allow correction for ascertainment of families, $L_f$ is the ratio of a numerator $N_f$, the contribution of a family f to the likelihood corrected by a denominator $D_f$ for ascertainment. The likelihood may be written as a function of the different parameters $x^j_G$, the risk for a mutation carrier (genotype G) of being affected in age-class j, given that he(she) was unaffected in the preceding class, and the corresponding $x^j_g$ for the non-carrier individuals (genotype g). This different $x^j_G$ are the estimated parameters and the $x^j_g$ were computed from, French population statistics (Benhamou et al, 1990; De Vathaire et al, 1996).

This method takes into account and corrects for three sources of bias due to selection criteria, namely (i) an excess of tumours before the age of 16, because the family was ascertained through an affected child, (ii) an excess of tumours before the age of 46, because at least one affected relative was requested, (iii) a deficit of individuals affected at a young age because most of the proband's parents and grandparents had reached a reproductive age without cancer. The denominator $D_f$ is the probability that the family f could be selected, i.e. the probability that there is a proband *and* that at least one relative is affected *and* that parents and grandparents were not affected before they reached a reproductive age.

A major advantage of this method is that it can use information on all family members including those in whom testing could not be performed. In the latter case, information is provided through their relationship with p53 mutation carriers. The risks are estimated separately for men and women, and the equality of risks between sexes is tested using the classic homogeneity test based on maximum likelihood (ML).

The denominator in the paper by Le Bihan et al (1995) was computed on the hypothesis that these three events were independent. In fact, events 1 and 3 are not totally independent and the program was modified to take this into account which, however, did not change the results (the accurate computation is available on request).

### RESULTS

### Families

Among the 2691 families investigated from January 1991 to May 1997, 295 fulfilled the selection criteria and gave their informed consent for DNA analysis. Twenty-seven children were excluded from the molecular analysis because they were affected by a genetic condition known to be linked to a gene other than p53: neurofibromatosis type 1, Beckwith–Wiedeman syndrome, WAGR syndrome, Gorlin syndrome and familial adenomatous polyposis. Finally, 252 'familial cases' and 16 'multiple tumour cases' fulfilling the selection criteria could be analysed. The diagnosis of cancer was confirmed by the medical and/or pathology records in 73% of the affected relatives.

Case distribution according to selection criteria is given in Table 1. Among familial cases, we have indicated whether they fulfilled the classic LFS criteria, or incomplete criteria (LFI defined as aggregation of typical LFS cancer lacking one of the classic criteria, no sarcoma in the proband, no first-degree relative affected or only one relative affected under the age of 45), or had multiple tumours without a family history. Table 2 presents the distribution of cases according to tumour type and date of diagnosis.

### Genotyping p53

For the first hundred samples p53 was genotyped by direct sequencing and subsequently by functional assay in yeast.

© 2000 Cancer Research Campaign

**Table 1** Cases according to selection criteria and family status and proportion of p53 germline mutation

| | Syndrome | Mutant | Wild-type | Total | Mutation (%) |
|---|---|---|---|---|---|
| Familial cases | LFS | 8 | 8 | 16 | 50.0 |
| | LFI | 4 | 62 | 66 | 6.1 |
| | Other | 4 | 166 | 170 | 2.3 |
| Multiple tumours[a] | | 1 | 15 | 16 | 6.2 |
| Total | | 17 | 251 | 268 | 6.3 |

[a] Without a family history. LFS: Li–Fraumeni syndrome; LFI: incomplete Li–Fraumeni syndrome.

**Table 2** Distribution of cases according to tumour type

| Diagnosis[a] | Proband studied before 1991 | Proband studied since 1991 | Total | p53 mutation |
|---|---|---|---|---|
| Soft tissue sarcoma | 40 | 14 | 54 | 6 (11%) |
| Osteosarcoma | 12 | 7 | 19 | 3 (15.8%) |
| Brain tumour | 18 | 25 | 43 | 4 (9%) |
| Adrenocortical carcinoma | 1 | 2 | 3 | 2 (66.7%) |
| Non-hodgkin's lymphoma | 27 | 11 | 38 | 0 |
| Ewing's sarcoma | 3 | 3 | 6 | 0 |
| Hodgkin's disease | 15 | 2 | 17 | 0 |
| Neuroblastoma | 33 | 13 | 46 | 2 (4%) |
| Nephroblastoma | 20 | 1 | 21 | 0 |
| Germ cell tumour | 12 | 4 | 16 | 0 |
| Other | 3 | 2 | 5 | 0 |
| Total | 184 | 84 | 268 | 17 |

[a] In case of multiple tumours the individuals are classified according to the first one.

**Table 3** Germline mutations in the p53 gene

| Family no. | Exon no. | Position codon no. | Mutation | Consequence | Status | Detection |
|---|---|---|---|---|---|---|
| 1975 | Intron 4/ Exon 5 | [126–132] | Addition G within splice acceptor site | → alternative splicing → In phase deletion [126–132] | De novo | Yeast+Sequence |
| 2471 | 4 | 125 | ACG→ACC | retention 109 bp from intron 4 | Inherited | Yeast+Sequence |
| 54 | 5 | 158 | CGC→GGC | Arg→Gly | Inherited | Sequence+Yeast |
| 11 | 5 | 175 | CGC→CAC | Arg→His | Inherited | Yeast+Sequence |
| 7[c] | 6 | deletion within 215 | AGT→AG | Frameshift→214 *bona fide* + 31 illegitimate residues | Inherited | Sequence |
| 151[b] | 7 | 248 | CGG→TGG | Arg→Trp | Inherited | Sequence+Yeast |
| 1128 | 7 | 245 | GGC→AGC | Gly→Ser | De novo | Yeast+Sequence |
| 2453 | 8 | [264–265] | del ACT | In phase deletion Leu#264 | Inherited | Yeast+Sequence |
| 231[b] | 8 | 273 | CGT→GGT | Arg→Gly | Inherited | Sequence+Yeast |
| 66 | 8 | 273 | CGT→CAT | Arg→His | Inherited | Yeast+Sequence |
| 867[b] | 8 | 282 | CGG→TGG | Arg→Trp | Inherited | Sequence+Yeast |
| 1497 | 8 | 282 | CGG→TGG | Arg→Trp | Inherited | Sequence+Yeast |
| 2454 | 8 | 273 | CGT→TGT | Arg→Cys | Inherited[a] | Yeast+Sequence |
| 2028 | 8 | 282 | CGG→CAG | Arg→Gln | Inherited | Yeast+Sequence |
| 147 | 8 | 273 | CGT→CTT | Arg→Leu | De novo | Sequence+Yeast |
| 641 | 8 | 281 | GAC→GTC | Asp→Val | De novo | Yeast+Sequence |
| 108 | 9 | 337 | CGC→CAC | Arg→His | Inherited | Sequence |

Yeast: mutation detectable by the yeast assay. [a]Inherited status highly probable. [b]Already described in Brugières et al (1993). [c]Already described in Stolzenberg et al (1994).

Seventeen p53 germline mutations were identified and are listed in Table 3. Eight mutations were detected by direct sequencing in the first set of 100 samples. These mutants were subsequently assayed in yeast: six scored positive, two negative, with the latter including mutation segregation in family no. 7 leading to a null allele (absence of mature mRNA in the cytoplasm), and mutation segregation in family no. 108 (Arg337His) which lies within the oligomerization domain but does not affect the transactivation capacity of p53. Nine mutations were detected among the 168 samples tested directly by the functional assay.

 *© 2000 Cancer Research Campaign*

**Table 4** Families with p53 mutation

| Family no. | Tumour type<br>Sex/Age at diagnosis (years) | Second tumour<br>Age at diagnosis (years) | Cancer in relatives<br>Age at diagnosis (years) | Type |
|---|---|---|---|---|
| 147 | ADCC (F/1) | RMS (1) | | Mult t |
| 641 | Osteo (M/18) | | MC breast (29) | Other |
| 1128 | Choroid plexus carcinoma (M/4) | | MA ovarian (30) | Other |
| 1975 | Medulloblastoma (M/10) | | MC neuroblastoma (7) | Other |
| 2028 | Neuroblastoma (F/1) | | MA lymphoma (44) | Other |
| 151 | RMS (M/2) | Osteo (20 y) | M breast (41)<br>sarcoma (52) | LFI |
| 867 | STS (M/14) | Osteo (28 y)<br>Lung (30 y) | S RMS (1)<br>brain (22)<br>breast (27) | LFI |
| 108 | ADCC (F/4) | | PU brain (33) | LFI |
| 2454 | Osteo (M/17) | | PGM bilateral breast (42) | LFI |
| 7 | RMS (M/3) | Osteo (11) | M breast (34)<br>B ADCC (11) | LFS |
| 231 | RMS (F/1) | | B brain stem tumour (4)<br>S ALL (6) | LFS |
| 54 | Osteo (M/11) | | M breast (29)<br>B RMS (2) | LFS |
| 11 | RMS (M/2) | Osteo (13) | M breast (29)<br>MA fibrosarcoma (6) | LFS |
| 1497 | Choroid plexus carcinoma (M/9) | | F lung carcinoma (40)<br>PA breast (33)<br>PC medulloblastoma (14)<br>sarcoma (22) | LFS |
| 2453 | RMS (F/7) | | B NHL (11)<br>S glioblastoma (25)<br>M breast (39)<br>MGM breast (30)<br>MU kidney sarcoma (25)<br>MA breast (43)<br>MC stomach (36)<br>MC breast (27)<br>MC bilateral breast (29)<br>MC breast (30)<br>N ADCC (3) | LFS |
| 66 | Neuroblastoma (M/5) | Osteo (11)<br>Osteo (13) | PGM NHL (42)<br>PA sarcoma (21)<br>PC ADCC (5)<br>PC brain (6) | LFS |
| 2471 | Ependymoma (M/2) | | M osteo (33)<br>MA breast (40) | LFS |

RMS: Rhabdomyosarcoma, ADCC: Adrenocortical carcinoma, STS: Soft tissue sarcoma, Osteo: Osteosarcoma, M: Mother, F: Father, B: Brother, S: Sister, PGF: Paternal grandfather, PGM: Paternal grandmother, MA: Maternal aunt, PA: Paternal aunt, PU: Paternal uncle, PC: Paternal cousin, MC: Maternal cousin, LFS: Li–Fraumeni syndrome, LFI: Incomplete Li–Fraumeni syndrome, Mult t: multiple tumours.

As shown in Table 1, eight mutations were detected in 16 of the LFS families (50%), four mutations in 66 LFI families (6.1%); one mutation was detected among 16 cases of multiple tumours without a family history (6.2%) and four mutations in ‘other’ cases of cancer aggregation (2.3%).

The tumours affecting probands shown to be p53 mutation carriers, as shown in Table 4, belong to the LFS spectrum, except for two cases of neuroblastoma. One of these children, however, developed a secondary osteosarcoma which does belong to the LFS spectrum. Among the child carriers with a brain tumour, there were two cases of choroid plexus carcinoma, a rare tumour already described in classic LFS (Jolly et al, 1994) and two cases of medulloblastoma which is not considered a classic LFS malignancy. Of the children having survived the first cancer, eight (five probands and three relatives) developed at least one second malignancy, two within the radiation field. Two to 11 (median 5) relatives were tested in families with inherited mutations. Eleven unaffected mutation carriers were detected, seven male individuals aged 22–62 (median 41) and four females aged 5–42.

Hereditary transmission of the mutant allele from one parent was demonstrated in 12 of the 17 mutations and was highly probable in one family in which the putative parent carrier had died and his affected relatives could not be tested (family no. 2454). In four cases, failure to demonstrate the presence of a mutation in any of the parents, led to the conclusion that the germline mutation had been acquired from a de novo mutated germ cell. In these four cases, genotyping, with two highly informative minisatellite markers, was consistent with true paternity status.

### Cancer risk estimates

The estimation of cancer risks was based on the 13 families in which the p53 mutation segregated. Nearly all cancer diagnoses were confirmed in these families (29/30 = 97%, among early-onset

© 2000 Cancer Research Campaign 

**Table 5** Risks of cancer in p53 mutation carriers according to age and sex

| Age (years) | Males | Females | Overall |
|---|---|---|---|
| 0–15 | 0.19 (0.09–0.35) | 0.12 (0.05–0.27) | 0.15 (0.08–0.27) |
| 16–45 | 0.27 (0.12–0.52) | 0.82 (0.42–0.96) | 0.54 (0.37–0.71) |
| >45 | 0.54 (0.07–0.95) | 1.00 (0.00–1.00) | 0.68 (0.14–0.97) |

cases and 2/4 = 50% among late-onset cases). The unconfirmed cases were excluded from the analysis.

The risks (probability of being affected in a given age-class, if unaffected in the preceding age category) are shown for each age-class in Table 5, where males and females are separated and then pooled. The risks in childhood are 19% for male and 12% for female children with an overall risk of 15%. The corresponding penetrance (probability of being affected before the end of the age-class) by age 16, 45 and 85 are respectively 19%, 41% and 73% in males, and 12%, 84% and 100% in females. In the older age-class (>45), only the p53 carriers who were not affected before 46 (i.e very few individuals and particularly females) provided information for these estimates. The lack of information in females leads to a large (0.00–1.00) confidence interval in this age-class. The penetrance is higher in females than in males ($P < 0.05$) and particularly in the 16–45 age-class, almost entirely because of breast cancer which represents 80% of all cancer cases in this age-class.

### DISCUSSION

The functional assay in yeast is a powerful method for the detection of mutations affecting the transactivation capacity of p53. Flaman et al (1995) demonstrated its ability to detect 90% of germline p53 mutations. In this study, the assay overlooked two mutations (not significantly different from the 10% reported): a mutation leading to a null allele and a mutation (Arg337His) lying within the oligomerization domain but obviously not affecting the transactivation capacity of p53. That the latter may represent a rare polymorphism cannot be ruled out, although this mutation has not been observed in more than 200 random alleles tested in this laboratory. An alternative missense mutation targeting codon 337 (Arg337Cys) has been reported in a LFL family. This mutation results in a protein which partially inhibits transactivation in the FASAY (Lomax et al, 1997). By contrast, a mutation (Leu344Pro) in close vicinity to this site, reported in a LFS family, gives rise to a totally defective protein in the FASAY (Varley et al, 1996). Our observation indicates that the loss of p53 transcriptional activity, at least in the FASAY, is not a prerequisite for bringing about hereditary predisposition to cancer within LFS.

In this study, we used far less stringent criteria than those defining LFS, in order to allow the detection of germline p53 mutations with low penetrance. In spite of these criteria, most of the mutations were found in families with a high aggregation of cancers. Nonetheless, unaffected carriers were observed and therefore the existence of families with low penetrance cannot ruled out. Varley et al (1999) also observed several unaffected carriers among the relatives of children with adrenocortical tumours not selected on family history. It is noteworthy that the detection of either unaffected carrier parents or de novo mutations in probands is favoured considering the poor prognosis of this disease. As a matter of fact, de novo mutations have occasionally been reported (Toguchida et al, 1992; Felix et al, 1993; McIntyre et al, 1994; Speiser et al, 1996). The selection criteria used in our study were a priori conducive to the detection of inherited cases. In spite of this, de novo mutations were observed in three of 188 index cases with only one affected relative and in one with multiple cancers (1.6%), indicating that de novo mutation is not a rare event. In fact, this is not unexpected considering the limited fecondity of the affected p53 mutation carriers.

It is remarkable that two de novo mutations were found among ‘familial cases’. Indeed, including affected first cousins for the selection of families, increases the probability of chance aggregation of sporadic cases. When such families are excluded from the selection criteria for mutation analysis, 233 ‘familial cases’ remain. The distribution of mutations among tumour types and types of family aggregations is virtually unchanged (data not shown).

Our study is consistent with previous reports which indicate that multiple tumours are frequent in germline p53 mutation carriers (Malkin et al, 1992; Eeles et al, 1993; Felix et al, 1993; Scott et al, 1993; Gutiérrez et al, 1994). Many of these tumours occurred in a radiation field, provoking concern about the risk associated with radiotherapy in these children. We did not exclude patients suspected of having therapy-induced malignancies in this study since radiation and familial factors were shown to be independent risk factors for the occurrence of second tumours (Kony et al, 1997).

As this sample represents a hospital-based population of childhood cancers, it is probably not strictly representative of all childhood cancers in the French population. Furthermore, there may be a deficit of tumours with a poor prognosis since blood samples were obtained only from alive children. This deficit is particularly marked in brain tumours as shown on Table 2. However, this should not impact on the cancer risk estimates since the method is independent of selection on tumour type. In addition, there could be a slight bias in favour of families with multiple affected members, but the robustness of the method has been proven with respect to this kind of bias when the risks are high (Le Bihan and Bonaïti-Pellié, 1994).

The confidence intervals of the penetrances estimated here are somewhat large, but this is due to the small number of families with p53 germline mutation. Nonetheless, it can be firmly stated that such penetrances are high, but not complete. The risk in childhood is slightly lower than previously published (Le Bihan et al, 1995) because of the correction of the computer program which in its first version tended to overestimate the risk in the first age-class. It is noteworthy that family ascertainment through childhood cancer may lead to select mutations with a particularly high risk of cancer at a young age. Interestingly, our estimates are quite consistent with those obtained from segregation analysis of families of children with soft tissue sarcoma (Lustbader et al, 1992; Strong et al, 1992) or through the follow-up of members of families with LFS (Garber et al, 1991). The older age category did not contribute much to the estimate of penetrance because of a limited amount of data in this age-class, and essentially the two age-classes (< 16 and 16–45) contribute to the likelihood of the hypotheses of respectively equal and different penetrance in males and females. The consequence of the difference between sexes is extremely important since the risk of developing cancer after childhood would mostly concern breast in women and would decline for men as they become older.

In conclusion, this study associates a complete family history investigation in a very large hospital-based population of children

 *© 2000 Cancer Research Campaign*

with a biological investigation in a subgroup selected with defined criteria. It demonstrates that only a small proportion of these cases can be ascribed to an inherited germline p53 mutations. It provides the first reliable estimations of cancer risks in p53 carriers and shows that these risks are very high. This study emphasises the existence of de novo mutations implying that germline mutations can be found in children with no or a limited family history.

### ACKNOWLEDGEMENTS

We are indebted to Brigitte Raynal for her help in paternity. We are grateful to Lorna Saint Ange for editing this manuscript. The collaboration of Dr Le Heug from the Centre Hospitalier de Nancy is gratefully acknowledged. This work was supported in part by ARC (contract no. 2011 to L.B.); la Ligue Nationale contre Le Cancer; la Caisse Nationale d'Assurance Maladie; La Caisse Nationale d'Assurance Maladie des Travailleurs Non Salariés; la Fondation de France; I'Institut Electricité Santé; I'Association ISIS (Association des parents d'enfants traités à I'Institut Gustave Roussy); I'Institut Gustave Roussy (CRC 92–8 to L.B.); Le Ministère de la Recherche et de l'Enseignement supérieur; SNECMA; INSERM; CNRS.

### REFERENCES

Birch JM, Hartley AL, Tricker KJ, Prosser J, Condie A, Kelsey AM, Harris M, Morris Jones PH, Binchy A, Crowther D, Craft AW, Eden OB, Evans DGR, Thompson E, Mann JR, Martin J, Mitchell ELD and Santibáñez-Koref MF (1994) Prevalence and diversity of constitutional mutations in the *p53* gene among 21 Li–Fraumeni families. *Cancer Res* **54**: 1298–1304

Brugières L, Gardes M, Moutou C, Chompret A, Meresse V, Martin A, Poisson N, Flamant F, Bonaïti-Pellié C, Lemerle J and Feunteun J (1993) Screening for germ line *p53* mutations in children with malignant tumors and a family history of cancer. *Cancer Res* **53**: 452–455

Eeles RA (1995) Germline mutations in the TP53 gene. *Cancer Surv* **25**: 101–124

Eeles RA, Warren W, Knee G, Bartek J, Averill D, Stratton MR and Blake PR (1993) Constitutional mutation in exon 8 of the *p53* gene in a patient with multiple primary tumors: molecular and immunohistochemical findings. *Oncogene* **8**: 1269–1279

Felix CA, Strauss EA, D'Amico D, Tsokos M, Winter S, Mitsudomi T, Nau MM, Brown DL, Leahey AM, Horowitz ME, Poplack DG, Costin D and Minna JD (1993) A novel germline *p53* splicing mutation in a pediatric patient with a second malignant neoplasm. *Oncogene* **8**: 1203–1210

Flaman J-M, Frebourg T, Moreau V, Charbonnier F, Martin C, Chappuis P, Sappino A-P, Limacher J-M, Bron L, Benhattar J, Tada M, Van Meir EG, Estreicher A and Iggo RD (1995) A simple *p53* functional assay for screening cell lines, blood, and tumors. *Proc Natl Acad Sci USA* **92**: 3963–3967

Frebourg T, Barbier N, Yan Y, Garber JE, Dreyfus M, Fraumeni J, Jr, Li FP and Friend SH (1995) Germline *p53* mutations in 15 families with Li–Fraumeni syndrome. *Am J Hum Genet* **56**: 608–615

Garber JE, Goldstein AM, Kantor AF, Dreyfus MG, Fraumeni JF, Jr and Li FP (1991) Follow-up study of 24 families with Li–Fraumeni syndrome. *Cancer Res* **51**: 6094–6097

Gutiérrez MI, Bhatia KG, Barreiro C, Spangler G, Schvartzmann E, Muriel FS and Magrath IT (1994) A de novo *p53* germline mutation affecting codon 151 in a six year old child with multiple tumors. *Hum Mol Genet* **3**: 2247–2248

Ishioka C, Frebourg T, Yan Y-X, Vidal M, Friend SH, Schmidt S and Iggo R (1993) Screening patients for heterozygous *p53* mutations using a functional assay in yeast. *Nat Genet* **5**: 124–129

Jolly KW, Malkin D, Douglass EC, Brown TF, Sinclair AE and Look TA (1994) Splice-site mutation of the *p53* gene in a family with hereditary breast-ovarian cancer. *Oncogene* **9**: 97–102

Knudson AG Jr (1971) Mutation and cancer. Statistical analysis of retinoblastoma. *Proc Natl Acad Sci USA* **68**: 820–823

Kony SJ, De Vathaire F, Chompret A, Shamsaldim A, Grimaud E, Raquin M-A, Oberlin O, Brugières L, Feunteun J, Eschwège F, Chavaudra J, Lemerle J and Bonaiti-Pellié C (1997) Radiation and genetic factors in the risk of second malignant neoplasms after a first cancer in childhood. *Lancet* **350**: 91–95

Le Bihan C and Bonaïti-Pellié C (1994) A method for estimating cancer risk in *p53* mutation carriers. *Cancer Detect Prev* **18**: 171–178

Le Bihan C, Moutou C, Brugières L, Feunteun J and Bonaïti-Pellié C (1995) Arcad: a method for estimating age-dependent disease risk associated with mutation carrier status from family data. *Genet Epidemiol* **12**: 13–25

Li FP, Fraumeni JF, Jr, Mulvihill JJ, Blattner WA, Dreyfus MG, Tucker MA and Miller RW (1988) A cancer family syndrome in 24 kindreds. *Cancer Res* **48**: 5358–5362

Lomax ME, Barnes DM, Gilchrist R, Picksley SM, Varley JM and Camplejohn RS (1997) Two functional assays employed to detect an unusual mutation in the oligomerisation domain of *p53* in a Li–Fraumeni like family. *Oncogene* **14**: 1869–1874

Lustbader ED, Williams WR, Bondy ML, Strom S and Strong LC (1992) Segregation analysis of cancer in families of childhood soft-tissue-sarcoma patients. *Am J Hum Genet* **51**: 344–356

Malkin D (1994) *p53* and the Li–Fraumeni syndrome. *Biochim Biophys Acta Rev Cancer* **1198**: 197–213

Malkin D, Jolly KW, Barbier N, Look AT, Friend SH, Gebhardt MC, Andersen TI, Borresen A-L, Li FP, Garber J and Strong LC (1992) Germline mutations of the p53 tumor-suppressor gene in children and young adults with second malignant neoplasms. *N Engl J Med* **326**: 1309–1315

Mclntyre JF, Smith-Sorensen B, Friend SH, Kassell J, Borresen A-L, Yan YX, Russo C, Sato J, Barbier N, Miser J, Malkin D and Gebhardt MC (1994) Germline mutations of the *p53* tumor suppressor gene in children with osteosarcoma. *J Clin Oncol* **12**: 925–930

Scott RJ, Krummenacher F, Mary J-L, Weber W, Spycher M and Müller H (1993) Inheritable *p53* mutation in a patient with multiple cancer and its significance for genetic counselling. *Schweiz Med Wochenschr* **123**: 1287–1292

Speiser P, Gharehbaghi-Schnell E, Eder S, Haid A, Kovarik J, Nenutil R, Sauter G, Schneeberger CH, Vojtesek B, Wiltschke CH and Zeillinger R (1996) A constitutional de novo mutation in exon 8 of the *p53* gene in a patient with multiple primary malignancies. *Br J Cancer* **74**: 269–273

Strong L, Williams W and Tainsky MA (1992) The Li–Fraumeni syndrome: from clinical epidemiology to molecular genetics. *Am J Epidemiol* **135**: 190–199

Toguchida J, Yamaguchi T, Dayton SH, Beauchamp RL, Herrera GE, Ishizaki K, Yamamuro T, Meyers PA, Little JB, Sasaki MS, Weichselbaum RR and Yandell DW (1992) Prevalence and spectrum of germline mutations of the *p53* gene among patients with sarcoma. *N Engl J Med* **326**: 1301–1308

Varley JM, McGown G, Thorncroft M, Cochrane S, Morrison P, Woll P, Kelsey AM, Mitchell ELD, Boyle J, Birch JM and Evans DGR (1996) A previously undescribed mutation within the tetramerisation domain of *TP53* in a family with Li–Fraumeni syndrome. *Oncogene* **12**: 2437–2442

Varley JM, Evans DGR and Birch JM (1997*b*) Li–Fraumeni syndrome: a molecular and clinical review. *Br J Cancer* **76**: 1–14

Varley JM, McGown G, Thorncroft M, Santibanez-Koref MF, Kelsey AM, Tricker KJ, Evans DGR and Birch JM (1997*a*) Germ-line mutations of *TP53* in Li–Fraumeni families: an extended study of 39 families. *Cancer Res* **57**: 3245–3252

Varley JM, McGown G, Thorncroft M, James LA, Margison GP, Forster G, Evans DG, Harris M, Kelsey AM and Birch JM (1999) Are there low-penetrance *TP53* alleles? Evidence from childhood adrenocortical tumors. *Am J Hum Genet* **65**: 995–1006

© 2000 Cancer Research Campaign
